# Pulmonary alveolar microlithiasis and interstitial pneumonitis: a case report of the west of Iran

**DOI:** 10.1051/bmdcn/2019090428

**Published:** 2019-11-14

**Authors:** Mazaher Ramezani, Zahra Aminparast, Masoud Sadeghi

**Affiliations:** 1 Molecular Pathology Research Center, Imam Reza Hospital, Kermanshah University of Medical Sciences Kermanshah Iran; 2 Students Research Committee, Kermanshah University of Medical Sciences Kermanshah Iran; 3 Medical Biology Research Center, Kermanshah University of Medical Sciences Kermanshah Iran

**Keywords:** Pulmonary alveolar microlithiasis, Case report, Interstitial pneumonitis

## Abstract

Pulmonary alveolar microlithiasis (PAM) is a rare disease with autosomal recessive inheritance. Herein, a 20-year-old lady referred to the hospital with a dry cough for two years. The chest X-ray findings were bilateral reticulonodular opacities in both lungs and honeycomb appearance suspicious for miliary tuberculosis and idiopathic pulmonary fibrosis. A wedge biopsy of lung showed that there were several intraalveolar laminated concretions in the pathology report compatible with pulmonary alveolar microlithiasis and interstitial infiltration of lymphocytes and neutrophils compatible with interstitial pneumonitis. PAM is a rare progressive disease with the production of microliths in pulmonary alveoli. The pathologist, radiologist, and clinician should be familiar with this entity for diagnosis and appropriate management. The family of the patient especially siblings must be evaluated for earlier diagnosis.

## 1. Introduction

Pulmonary alveolar microlithiasis (PAM) is a rare disease with autosomal recessive inheritance which affects mainly the patients between 20s and 40s [1–4]. The pathogenesis of the disease is known to be a mutation in the gene which encodes sodium-dependent phosphate cotransporter. Accumulation of phosphorous from a degraded surfactant in the pulmonary alveoli makes a nidus for microliths or stone lungs [1, 2, 5–9]. The stones are mainly made of calcium phosphate with a lamellated or psammoma-body appearance in histologic sections [1, 2]. In the early phase, most of the patients are asymptomatic. As the disease progresses, the presentation will be a dry cough, dyspnea on exertion, cyanosis, chest pain, hemoptysis or pneumothorax [1, 2, 5, 10]. Finally, the disease will progress to corpulmonale and respiratory failure [2]. Approximately, one thousand cases are reported in the world right now and most of the articles in the literature are case reports [2]. Diagnosis is possible by imaging, but a definite diagnosis requires a pulmonary biopsy [5, 11]. PAM is in differential diagnosis with other disorders especially pulmonary tuberculosis and may be prescribed unnecessary antitubercular therapy mainly in endemic areas [2, 12]. Herein, we report a rare case of PAM associated with interstitial pneumonitis with the classic presentation which is diagnosed in histopathology examination of lung biopsy in the west of Iran.

## 2. Case report

**Fig. 1:**
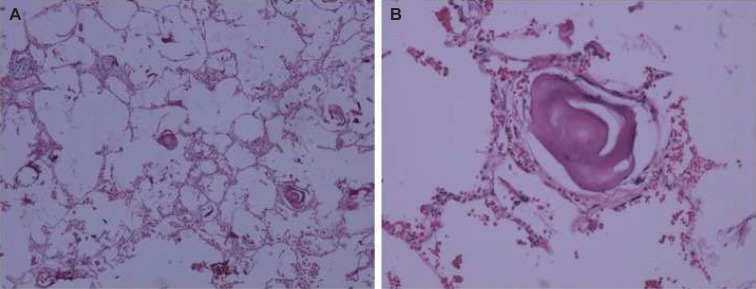
Pulmonary Alveolar Microlithiasis showing microliths in alveolar spaces. (Hematoxylin-Eosin staining with magnifications of A ×40 and B ×200)

A 20-year-old lady referred to the hospital with a chief complaint of dry cough since 2 years ago on 31^st^ December 2018 to the department of thoracic surgery for lung biopsy. Severe cough attack lasted 5-10 minutes each time. Attacks were more severe for 3-4 days. Past medical history, drug history, and family history were unremarkable. There was no dyspnea or respiratory distress, fever and chills, nausea or vomiting. The physical examination was unremarkable including stable vital signs. Temperature: 37°C, Pulse rate: 78/min, Respiratory rate: 19/min and Blood pressure: 110/60 mmHg. Chest wall was symmetric with soft abdomen. No heart murmur or abdominal tenderness was noted. The general condition was good. The chest X-ray findings were bilateral reticulonodular opacities in both lungs (Pneumonia) and honeycomb appearance (Bronchiectasis) suspicious for miliary tuberculosis and idiopathic pulmonary fibrosis. Opacity and mucosal thickness in maxillary sinuses suggested sinusitis. The ultrasound revealed diffuse opacities in both lungs. Meanwhile, bilateral axillary lymphadenopathy measuring 11-13 mm with reactive appearance was present. Spiral Computed Tomography (CT) scanning without contrast of lung and mediastinum revealed evidence of diffuse opacities of reticulonodular pattern in both parenchymas suggestive of miliary tuberculosis and less probable military metastasis. A few foci of linear fibrosis and air trapping in both lungs’ parenchyma were also noted. Clinically, sarcoidosis was also in differential diagnosis. Bronchoalveolar lavage demonstrated no evidence of malignancy and evidence of chronic inflammation and calcification. The lab data including urea (17 mg/*dl*), creatinine (0.7 mg/*dl*), Na (137 mEq/l), K (4 mEq/l), and blood sugar (130 mg/*dl*) were within normal limits. Prothrombin Time (PT): 13.7 second, International Normalized Ratio (INR): 1, Partial Thromboplastin Time (PTT): 24 second and liver function tests were also within normal limits. White blood cell counts were 18.3 and 16.2 ×10^3^/microliter at two consequent days. Neutrophils were about 85%. Hemoglobin and platelets were unremarkable. The left lateral thoracotomy with a primary diagnosis of idiopathic pulmonary fibrosis was done and wedge biopsy from the left lower lobe was taken. The specimen consisted of a tan-brownish piece of tissue measuring 2.5 × 2 × 0.5 cm. The pathology report showed several intra alveolar laminated concretions compatible with PAM. The lung tissue showed interstitial infiltration of lymphocytes and neutrophils compatible with interstitial pneumonitis (Figure 1)

## 3. Discussion

PAM is rare, but most cases are from Asia and Europe. In Asia, the total number of cases in the review of Castellana *et al.* [2] from Iran was 26 ranking 5th after Turkey, China, Japan and India. The disease is slightly more prevalent in men [2]. Although the disease is rare in Africa, Zaghba *et al.* [13] reported a 42-year-old African lady with PAM by chest X-ray, chest CT scan, and transbronchial biopsy. These tools were also used by researchers of Turkey for confirmation of their case of PAM [14].

Devine et al. [15] reported a 56-year-old Turkish man of PAM presented with mild dyspnea and a lifelong history of chronic lung disease. Despite pneumothorax, his patient was asymptomatic at rest. Familial cases of PAM are reported in the literature with a positive family history in 37.2% of cases [1, 2]. One study [16] from China reported 3 cases of PAM, two of them had consanguinity. PAM has been seen in all age groups, but most cases are between 20 and 40 [2] however, stamatopoulos and the colleagues reported a 63-year-old man with a positive family history as an unusually late-onset disease [17]. Gupta *et al.* [18] diagnosed their case of PAM by adding a new pattern to radiologic pictures of PAM, as “dense confluent calcifications caused consolidation of the lungs” on high resolution computed tomographic scan of the lung. Two studies [19, 20] reported PAM cases with Clinico-Radiological dissociation that one study [19] showed a silent clinic with extensive changes on imaging and biopsy, whereas another study [20] confirmed the diagnosis by bronchoscopic alveolar lavage and demonstration of calcospherites.

Some researchers believe that radiology is enough, for diagnosis of PAM, when findings are typical in an asymptomatic patient. Differential diagnosis of an asymptomatic patient with chest X-ray showing dense micronodular and ground glass opacities, is mainly miliary tuberculosis, pulmonary alveolar proteinosis, sarcoidosis, resolved varicella pneumonia, metastatic calcification, pneumoconiosis and amyloidosis [1, 2]. Patchy inflammation and pulmonary fibrosis are frequently seen in PAM [1]. Present case had interstitial inflammation mainly of lymphocytes. Others reported lymphocytic interstitial pneumonitis in PAM, but it may be an association by chance [1]. Long-term prognosis is poor and the disease may culminate in respiratory failure [1]. Smoking, inflammation, and cold temperature may deteriorate the patient’s condition [2]. Etidronate as a bisphosphonate, steroid hormones and repeated lavage are controversial medical treatments. Supplemental oxygen therapy and vaccination against pneumococcus and influenza are necessary for patients. Lung transplanted patients had no recurrence of the disease [1]. Gene therapy may be a promising treatment in the future [2].

## 4. Conclusions

PAM is a rare progressive disease with the production of microliths in pulmonary alveoli. This entity has the characteristic radiologic picture in a silent clinical background and can be proven cytologically or histologically by bronchoalveolar lavage or lung biopsy, respectively. The pathologist, radiologist, and clinician should be familiar with this entity for diagnosis and appropriate management. The patient’s family especially siblings must be evaluated for earlier diagnosis.
